# Diagnostic Performance of Different AI Tools for Chest Radiography: A CT-Anchored Per-Abnormality Analysis in a Tertiary Referral Center

**DOI:** 10.3390/diagnostics16193139

**Published:** 2026-09-27

**Authors:** Selin Ardalı Düzgün, İlke Taşçı, Atahan Tamtürk, Nuri Saraç, Melih Karadağ, Mustafa Ege Şeker, Deniz Köksal, Sevinç Sarınç, Meltem Gülsün Akpınar, Figen Başaran Demirkazık, Gamze Durhan

**Affiliations:** 1 Department of Radiology, Faculty of Medicine, Hacettepe University, Ankara 06100, Turkey; selin.ardali@gmail.com (S.A.D.); ilketascii@gmail.com (İ.T.); atatamturk99@gmail.com (A.T.); nurisarac1998@gmail.com (N.S.); melihkaradag5899@gmail.com (M.K.); meltemg@hacettepe.edu.tr (M.G.A.); fdemirka@gmail.com (F.B.D.); 2 Department of Radiology, School of Medicine and Public Health, University of Wisconsin-Madison, Madison, WI 53705, USA; mseker@wisc.edu; 3 Department of Chest Diseases, Faculty of Medicine, Hacettepe University, Ankara 06100, Turkey; dckoksal@gmail.com (D.K.); sevincsarinc@gmail.com (S.S.)

**Keywords:** artificial intelligence, thorax, X-ray, computed tomography

## Abstract

**Background/Objectives**: Artificial intelligence (AI) increasingly supports chest radiograph interpretation, but per-abnormality comparisons of commercial tools using CT-anchored reference standards remain limited. We compared two commercial AI tools using a CT-anchored, CXR-targeted reference framework. **Methods**: We retrospectively reviewed chest radiographs obtained between 30 October 2023 and 10 October 2024. Each radiograph was paired with CT performed within 14 days or within 2 days for rapidly progressive findings. Seven abnormalities across five analysis categories were evaluated: fracture, nodule, pleural effusion, pneumothorax, and airspace disease (edema, atelectasis, opacity/consolidation). Radiologists first confirmed abnormalities on CT then assessed their visibility on the paired radiograph. Outputs from vendors A and B were recorded. Sensitivity was calculated for CT-confirmed, radiographically visible abnormalities; specificity, precision, and accuracy were calculated within the same detectability framework. Vendors were compared using McNemar’s test. **Results**: The cohort included 1707 radiographs from 1680 patients (mean age, 60.3 ± 16.5 years; 50.5% male). Of 3573 CT-confirmed abnormalities, 1981 (55.4%) were radiographically visible and formed the primary reference-positive set. Vendor A had higher sensitivity for fractures, nodules, and pleural effusions (all *p* < 0.001), whereas vendor B had higher specificity for these findings (all *p* < 0.05). For pneumothorax, vendor B showed numerically higher sensitivity but lower specificity and accuracy; case numbers were limited, and precision was low for both tools. No significant differences were observed for harmonized airspace disease. Overall specificity was high, while sensitivity and precision varied across abnormalities. **Conclusions**: Both tools showed abnormality-specific strengths and limitations, supporting tailored implementation and real-world clinical validation.

## 1. Introduction

Chest X-ray (CXR) remains the initial imaging modality for evaluating thoracic pathologies and plays a central role in clinical practice due to its low cost, low radiation dose, and widespread availability [1,2,3]. However, several factors limit its diagnostic utility, including the shortage or uneven distribution of experienced radiologists, increasing workload pressures that constrain reporting times, and in some cases, the absence of routine reporting protocols. These challenges can contribute to missed or delayed diagnoses.

In response, artificial intelligence (AI) tools have emerged as promising solutions to augment CXR interpretation. Particularly in thoracic radiology, AI applications have gained significant attraction, leading to a proliferation of commercial algorithms for CXR analysis [4]. Numerous studies have shown that AI can aid in differentiating normal from abnormal radiographs, improve diagnostic accuracy and sensitivity, enhance the performance of both radiologists and non-radiologist physicians, and reduce reporting times [5,6,7,8,9,10].

Despite these advances, most prior studies have relied on radiologist reports or expert consensus as the reference standard [5,6,8,11,12,13]. This approach is inherently limited by human error and interobserver variability, which is notably high in CXR interpretation [14,15,16]. CT can provide a stronger anchor for confirming abnormalities; however, CT-informed reference frameworks in chest radiography may be defined differently. Prior work has shown that performance differs when all CT-positive findings are used as the endpoint versus when only CT-confirmed abnormalities that are radiographically visible on CXR are considered reference-positive [17]. In addition, CT-informed studies have generally focused on case- or exam-level analyses rather than abnormality-level head-to-head comparisons [7,18,19].

Given the increasing availability of diverse AI tools for CXR interpretation, there is a clear need for head-to-head comparisons of their diagnostic performance across various abnormality types. Such comparative analyses are critical for understanding tool-specific strengths and limitations in real-world clinical settings [1,2,3].

This study aims to evaluate and compare the per-abnormality performance of two commercially available AI software tools for CXR interpretation using a CT-anchored, CXR-targeted reference framework focused on radiographically detectable CT-confirmed abnormalities.

## 2. Materials and Methods

### 2.1. Study Population

Consecutive CXRs obtained between 30 October 2023 and 10 October 2024, and analyzed by two different commercially available AI software tools, were retrospectively reviewed. Eligible cases included posterior–anterior (PA) or portable anterior–posterior (AP) CXRs of patients aged 18 years or older who underwent a chest CT scan within 14 days of the X-ray. For abnormalities that may change rapidly—pleural effusion, consolidation, pneumothorax, and pulmonary edema—the acceptable time interval between X-ray and CT was restricted to 2 days. All imaging data were routinely acquired chest radiographs and CT examinations performed as part of standard patient care.

If a patient had multiple CXRs, each CXR was included only if it corresponded to a distinct CT examination. Lateral radiographs (as neither AI tool supports their use), non-diagnostic images (e.g., due to motion artifacts or incomplete field of view), and CXRs from patients who underwent trauma or thoracic surgery between the CXR and CT were excluded.

### 2.2. Data Collection

CXRs were acquired using GE Definium 8000 (GE Healthcare, Chicago, IL, USA), Fujifilm FDR Smart FGXR-68 S (Fujifilm Corporation, Tokyo, Japan) and Siemens Multix Fusion Max (Siemens Healthineers, Erlangen, Germany) systems. Chest CT examinations were performed using multidetector scanners (Somatom Force, Somatom Perspective, Somatom Definition and Sensation 16, Siemens Healthineers, Erlangen, Germany; LightSpeed 16 and Optima CT 540, GE Healthcare, Chicago, IL, USA), with/without intravenous contrast administration based on clinical indication, and reconstructed at slice thicknesses of 1–1.5 mm.

### 2.3. AI Software Used in the Study

This study evaluated two CXR AI solutions (vendor A and B). Vendor A is an MDR CE-marked, deep learning–based CXR analysis tool. According to the manufacturer, it was developed using a training dataset of more than 300,000 posteroanterior and anteroposterior CXRs annotated with bounding boxes by expert radiologists. The algorithm uses a convolutional neural network based on a U-Net architecture and is designed to detect and localize consolidation, undefined lung opacity, pulmonary edema, fracture, nodule, atelectasis, pleural effusion, and pneumothorax.

Vendor B is an MDR CE-marked and FDA-cleared, deep learning–based CXR analysis tool. According to the manufacturer, it was developed using U-Net-based convolutional neural networks trained on approximately 4 million CXRs annotated by expert radiologists. In the present study, the outputs evaluated for vendor B were consolidation, opacity, fracture, nodule, pleural effusion, and pneumothorax.

For this study, outputs from both vendors were evaluated using the manufacturers’ default operating thresholds, without site-specific recalibration.

Both software tools were integrated with picture archiving and communication systems (PACS) (Centricity, GE Healthcare, Chicago, IL, USA) and received CXR images directly from the PACS environment. No additional preprocessing was performed. Each algorithm automatically analyzed CXR and returned the results to the PACS in the form of DICOM secondary capture images for storage. No clinical information or reference standard results were available to either algorithm. Since eligibility criteria required a valid output from both AI tools and a paired CT examination, no index test or reference standard data were missing in the analysis set.

### 2.4. Abnormality-Based X-Ray Evaluation and CT-Anchored Reference Framework

A thoracic radiologist with 6 years of experience, together with at least one of four senior radiology residents participating in the study, reviewed all chest CT and paired CXR examinations in consensus. In cases of diagnostic uncertainty, a second thoracic radiologist with 10 years of experience was consulted, and a final decision was reached. Because visibility adjudication was performed in consensus rather than independently, interobserver agreement statistics were not calculated.

The study was designed to evaluate the radiographic detectability of CT-confirmed abnormalities on CXR rather than strict CT-defined disease detection. Chest CT scans performed within 14 days of the corresponding CXR (or within 2 days for findings prone to rapid progression, such as pleural effusion, consolidation, pneumothorax, and pulmonary edema) served as the anchoring imaging modality to confirm abnormalities.

The CT scans were first reviewed to identify the presence or absence of the seven predefined abnormalities: atelectasis, pulmonary edema, fracture, nodule, opacity/consolidation, pleural effusion, and pneumothorax. Given the inherent limitations of chest radiography—such as its projectional nature, limited soft tissue contrast, and the superimposition of anatomical structures—some CT-confirmed abnormalities may not be detectable on CXR. Therefore, the paired CXRs were reviewed to determine whether each CT-confirmed abnormality had a corresponding radiographic manifestation on the index radiograph. Findings were categorized as CXR-visible or CXR-inapparent. CXR-inapparent findings included those that are not reliably detectable on X-ray due to inherent technical limitations, as well as findings with no potential clinical impact, such as nodules smaller than 6 mm, linear or subsegmental atelectasis, small-volume pleural effusions, and findings obscured by overlying structures or adjacent dominant abnormalities. Nodules smaller than 6 mm were categorized as CXR-inapparent, as nodules of this size are often not reliably detectable on CXRs, even when present on CT. A phantom-based study reported radiologists’ sensitivity for detecting lung nodules on CXR was 82% at 10 mm, 66% at 8 mm, and 14.3% at 5 mm, indicating a sharp detectability decline within this range [20]. Therefore, a 6 mm cutoff was used to avoid overestimating false negatives. This cutoff is also consistent with clinically relevant size thresholds used in screening systems such as Lung-RADS and Fleischner Society guidelines, although these address clinical management rather than radiographic visibility [21,22].

For the primary analysis, only CT-confirmed abnormalities adjudicated as radiographically visible on the paired CXR were considered reference-positive. CT-positive findings deemed radiographically inapparent on CXR were handled as follows: they were excluded from the reference-positive set and therefore from the primary sensitivity denominator. Furthermore, they were not reclassified as reference-negative and contributed no true negatives; an algorithm output corresponding to such a finding was not counted as a false positive because the abnormality was genuinely present on CT. These instances were therefore removed from the analysis set entirely rather than assigned to either reference category [17,23]. A flow diagram summarizing this process is provided in Supplementary Figure S1.

Subsequently, for each abnormality, the radiologists assessed the outputs of both AI tools and recorded whether the software provided a true positive (TP), false positive (FP), false negative (FN), or true negative (TN) result. The radiologists were blinded to the AI outputs during CT review and CXR visibility assessment. The outputs of both AI tools were reviewed only after the reference labeling was completed.

For example, if a CT-confirmed nodule had a corresponding radiographically visible manifestation on the paired CXR and was annotated by the AI tool, it was marked as a true positive. If the AI failed to detect the visible nodule, it was marked as a false negative. If the AI flagged a nodule without a corresponding CT-confirmed radiographically visible lesion on the paired chest radiograph, it was considered a false positive.

In this study, performance analyses of the AI software tools were conducted on a per-abnormality basis. If a patient had multiple abnormalities of the same type in different anatomic regions, each was evaluated separately. When different abnormalities overlapped in the same region, each was first assessed independently; however, if one abnormality obscured the radiographic manifestation of another, the obscured finding was categorized as CXR-inapparent for the purpose of this CXR-targeted analysis.

A total of seven findings were evaluated in this study. Four findings—nodule, pleural effusion, pneumothorax, and fracture—were common to both AI tools and directly matched based on equivalent annotation labels. However, the categories of pulmonary edema and atelectasis, available in vendor A, were not explicitly represented in vendor B. For fair vendor-to-vendor comparison, vendor-specific airspace outputs (atelectasis, edema, opacity, and consolidation for vendor A, and opacity and consolidation for vendor B) were harmonized into a single comparison category defined as airspace disease.

### 2.5. Statistical Analysis

No formal sample size calculation was performed, since all eligible consecutive examinations were included. All analyses were conducted using Python 3.11 (Python Software Foundation, Wilmington, DE, USA) with the pandas, numpy, and statsmodels libraries. We performed an abnormality-based, head-to-head comparison of two AI tools within a CT-anchored, CXR-targeted reference framework. Performance metrics included sensitivity, specificity, precision (positive predictive value), and accuracy. Because the analysis set for each abnormality comprised predominantly reference-negative instances, accuracy closely tracks specificity. Ninety-five percent confidence intervals (CIs) for proportions were calculated using the Wilson score method. A two-tailed *p*-value < 0.05 was considered statistically significant.

For the primary analysis, sensitivity was calculated using only CT-confirmed abnormalities adjudicated as radiographically visible on the paired CXR. CT-positive findings deemed radiographically inapparent were excluded from the primary sensitivity denominator because the study endpoint was radiographic detectability on CXR rather than strict CT-defined disease detection. Specificity was calculated using all CT-negative cases for the corresponding abnormality. Precision and accuracy were calculated within the primary analysis set, which comprised CT-negative cases together with CT-confirmed CXR-visible positive cases.

Paired comparisons between algorithms were conducted using the exact two-sided McNemar test, limited to lesions for which both algorithms generated valid predictions. McNemar’s test was specifically selected as we performed an abnormality-based, head-to-head comparison of two AI tools on the exact same CXRs, and the diagnostic outputs are dependent. Rather than simply comparing overall accuracy, McNemar’s test accounts for this correlated data by evaluating the discordant pairs—instances where one algorithm correctly identified the abnormality while the other failed [24]. This provides a rigorous assessment of whether the difference in diagnostic performance between the two tools is statistically significant. McNemar’s test was not applicable to precision, because the two algorithms generate positive calls on overlapping but non-identical sets of instances and the denominators of the two positive predictive values therefore differ. Precision was instead compared using the regression approach for paired designs described by Leisenring et al., in which each algorithm-positive call contributes a single observation with the reference status as the outcome and the algorithm as the covariate, estimated with a marginal logistic model with an independence working correlation structure and a cluster-robust variance estimator with the patient as the clustering unit [25].

Given the exploratory nature of evaluating these AI tools across a diverse range of clinical abnormalities, no adjustments were made for multiple comparisons. Accordingly, the reported *p*-values are unadjusted and should be interpreted descriptively. While this approach avoids inflating Type II errors, performing multiple head-to-head comparisons inherently increases the risk of Type I errors [26]. As a sensitivity analysis, Bonferroni and Holm–Bonferroni corrections were additionally applied across the 20 primary abnormality-by-metric comparisons, corresponding to five abnormality categories and four performance metrics. Corrected *p*-values are reported in Supplementary Table S1 to clarify which findings remained robust after adjustment and which were sensitive to multiple-comparison correction.

Because the unit of analysis was the abnormality instance rather than the patient, and a given patient could contribute more than one instance of the same abnormality type, the paired observations entering the McNemar comparison may not be fully independent. Two additional analyses were therefore performed to assess the influence of within-patient clustering. First, all comparisons were repeated using a marginal logistic model with an independence working correlation structure and a cluster-robust variance estimator with the patient as the clustering unit, with the algorithm entered as a within-cluster factor; this provides the clustered analogue of the paired McNemar comparison. Second, a patient-level analysis was performed in which each patient contributed a single observation per abnormality category, being classified as reference-positive if at least one CT-confirmed, radiographically visible abnormality of that category was present and as test-positive if the algorithm flagged that abnormality on the paired radiograph; patients whose only CT-confirmed findings of a category were adjudicated as radiographically inapparent were excluded from that category, consistent with the primary reference framework. Within each category, observations in the patient-level analysis are independent and were compared using the exact McNemar test. Results of both analyses are presented in Supplementary Tables S2 and S3.

A subgroup analysis stratified by radiographic projection was performed to assess whether the relative performance of the two tools was affected by the technical differences between PA and portable AP radiography. Each abnormality instance was assigned the projection of the radiograph on which it was assessed. This analysis was exploratory, and *p*-values are unadjusted; results are presented in Supplementary Table S4.

This article adheres to the STARD-AI statement, and the checklist is provided as Supplementary Table S5 [27].

## 3. Results

### 3.1. Study Cohort

The study included 1707 CT-paired CXR exams from 1680 unique patients (mean age ± SD = 60.3 ± 16.5 years; 50.5% male). Among included CXR exams, 1378 (80.7%) were PA and 329 (19.3%) were portable AP. The median interval between CXR and paired CT was 1 day (interquartile range [IQR]: 5 days). The median intervals were also similar for the nodule and fracture subgroups (median: 1 day, IQR: 5 days).

### 3.2. CT-Anchored Reference Framework and Chest X-Ray Visibility

A total of 13,158 abnormality instances across seven pathology categories were assessed within the CT-anchored reference framework. Chest CT was used to confirm abnormality presence and type, and the paired CXR was then adjudicated for radiographic visibility.

Among these, 3573 abnormalities were confirmed on chest CT scans. Of these CT-confirmed findings, 1592 (44.6%) were classified as radiographically inapparent on CXR, while 1981 (55.4%) were deemed radiographically visible and constituted the reference-positive set for the primary per-abnormality analysis. The remaining 9585 findings showed no corresponding abnormality on CT and served as reference-negative cases. Accordingly, the reported sensitivity values reflect detection of CT-confirmed radiographically visible abnormalities on chest radiography rather than strict CT-based disease sensitivity. Table 1 presents the distribution of abnormalities according to CT confirmation and CXR visibility across the abnormality categories.

Among CT-confirmed cases of fracture, pleural effusion, and pneumothorax, the proportion of findings that were inapparent on CXR was 46% (*n* = 109), 28.5% (*n* = 192), and 23% (*n* = 6), respectively. Portable CXRs accounted for 34%, 46.3%, and 33.3% of these non-visible cases, respectively. For pleural effusion, the most common cause of non-visibility was small volume (*n* = 118, 61.5%). All six pneumothoraces missed on CXR were attributed to minimal air volume.

Of the 783 nodules detected on CT, 588 (75%) were not visible on CXR; among these, 14% were from portable exams. The main limiting factor was small nodule size: 498 nodules (84.7%) were ≤1 cm, and 433 (73.6%) were <6 mm. Other contributing factors included location—such as apical, central, peribronchovascular, subpleural, perifissural, retrocardiac, and costophrenic regions (*n* = 57, 9.7%)—ground-glass density (*n* = 12, 2.0%), obscuration by the diaphragm or osseous structures (*n* = 14, 2.4%), and overlap with consolidation or atelectasis (*n* = 7, 1.2%).

Among the 1854 CT-confirmed abnormalities harmonized under the category of airspace disease, 697 (37.6%) were inapparent on CXR. Of these inapparent findings, 20 (2.9%) were pulmonary edema, 96 (13.8%) were opacities, and 581 (83.4%) were atelectasis. Portable CXRs accounted for 30%, 29.2%, and 23.4% of these, respectively. For atelectasis, the primary reason for non-visibility was linear or subsegmental atelectasis (*n* = 555, 95.5%). Additional causes included passive atelectasis (*n* = 17, 2.9%), adjacency to consolidation or mass (*n* = 4, 0.7%), and unfavorable location (*n* = 5, 0.86%). Regarding opacities inapparent on CXR, ground-glass opacities accounted for the majority (*n* = 42, 43.8%), followed by mild centrilobular opacities (*n* = 29, 30.2%), location-related limitations (*n* = 10, 10.4%), and obscuration by other abnormalities (*n* = 15, 15.6%). Within the harmonized airspace disease category, opacities/consolidations accounted for 56.6% of the findings adjudicated as visible on CXR, followed by atelectasis (29.6%) and edema (13.8%).

### 3.3. Abnormality-Based Comparison of Performance Metrics Between Two Different AI Tools

#### 3.3.1. Fracture

Vendor A showed significantly higher sensitivity and accuracy compared to vendor B. Both algorithms demonstrated very high specificity (98.9% and 99.6%, respectively). Vendor B showed higher specificity on unadjusted analysis (*p* = 0.035); however, this finding did not remain significant after Bonferroni or Holm–Bonferroni correction and should therefore be interpreted cautiously. Conversely, the differences in sensitivity and accuracy remained statistically significant after correction (*p* < 0.05). The precision of both algorithms was similar (*p* = 0.92) (Table 2, Supplementary Table S1) (Figure 1).

#### 3.3.2. Nodule

In nodule detection, vendor A showed significantly higher sensitivity than vendor B (*p* < 0.001) but slightly lower precision (*p* = 0.47) and accuracy (*p* = 0.40), which did not reach statistical significance. Also, vendor B achieved significantly higher specificity compared to vendor A (*p* < 0.001). Both the sensitivity and specificity differences remained significant after Bonferroni and Holm–Bonferroni correction (*p* < 0.05) (Table 2, Supplementary Table S1) (Figure 2 and Figure 3).

#### 3.3.3. Pleural Effusion

For pleural effusion, vendor A demonstrated significantly higher sensitivity compared to vendor B (*p* < 0.001). The accuracy for pleural effusion was equal between the two algorithms (*p* = 1.000). However, the specificity and precision of vendor B were significantly higher than those of vendor A (both *p* < 0.001). Following Bonferroni and Holm–Bonferroni correction, the differences in sensitivity, specificity, and precision remained significant (*p* < 0.05) (Table 2, Supplementary Table S1).

#### 3.3.4. Pneumothorax

In the detection of pneumothorax, vendor B showed numerically higher sensitivity than vendor A, although this difference did not reach statistical significance (*p* = 0.063). Precision was very low for both algorithms; however, vendor B had significantly lower precision than vendor A (4% versus 38%, *p* < 0.001). Vendor A demonstrated significantly higher specificity and accuracy than vendor B (both *p* < 0.001). The specificity, accuracy, and precision differences remained significant after Bonferroni and Holm–Bonferroni corrections (*p* < 0.05) (Table 2, Supplementary Table S1) (Figure 4).

#### 3.3.5. Airspace Disease

For the harmonized airspace disease category, there were no statistically significant differences between the two vendors (sensitivity: *p* = 0.28; specificity: *p* = 0.62; precision: *p* = 0.41; accuracy: *p* = 0.23) (Table 2, Supplementary Table S1) (Figure 5).

#### 3.3.6. Sensitivity Analyses for Within-Patient Clustering

Accounting for within-patient clustering did not materially alter the head-to-head comparisons. In the cluster-robust re-analysis (Supplementary Table S2), all differences that were statistically significant in the primary analysis remained significant and in the same direction, and all non-significant comparisons remained non-significant, with one exception: the difference in pneumothorax sensitivity reached nominal significance (60.0% versus 85.0%, *p* = 0.02) whereas the exact paired test did not (*p* = 0.063). Given only 20 reference-positive pneumothoraces, the asymptotic clustered estimate is unstable, and this comparison is not considered significant. In the patient-level analysis (Supplementary Table S3), which included 1625 patients for fracture, 1130 for nodule, 1565 for pleural effusion, 1653 for pneumothorax and 1316 for airspace disease, vendor A again showed higher sensitivity for fracture (78.1% versus 23.3%), nodule (76.9% versus 46.3%) and pleural effusion (91.3% versus 60.5%) (all *p* < 0.001), and vendor B higher specificity for nodule (95.3% versus 90.3%) and pleural effusion (95.5% versus 87.1%) (both *p* < 0.001). For pneumothorax, vendor A retained significantly higher specificity, precision, and accuracy (all *p* < 0.001), and the sensitivity difference again did not reach significance (*p* = 0.063). For harmonized airspace disease, modest differences emerged at the patient level that were not apparent at the lesion level, with higher sensitivity for vendor A (79.6% versus 74.9%, *p* = 0.028) and higher specificity and precision for vendor B (93.7% versus 89.3%, *p* = 0.002; 93.4% versus 89.9%, *p* = 0.005); these exploratory findings follow the same sensitivity–specificity trade-off observed for the other abnormality categories.

The sensitivity and specificity of each vendor for different abnormalities are summarized in Table 3. The confusion matrix of both algorithms for different X-ray abnormalities is given in Table 4.

#### 3.3.7. Subgroup Analysis by Radiographic Projection

The relative performance of the two tools was broadly consistent across projections (Supplementary Table S4). Vendor A retained significantly higher sensitivity for fracture and pleural effusion in both the PA and the portable AP stratum, and significantly higher specificity, precision, and accuracy for pneumothorax in both. Comparisons that lost significance in the portable AP stratum involved small reference-positive subgroups rather than a reversal of effect. Absolute performance was lower on portable AP radiographs for both tools: specificity for airspace disease was 94.8% on PA versus 82.1% on portable AP radiographs for vendor A, and 94.4% versus 86.1% for vendor B (both *p* < 0.001). One projection-dependent difference was observed between the tools. The sensitivity of vendor B for pleural effusion was substantially lower on portable AP than on PA radiographs (44.7% versus 72.3%, *p* < 0.001), whereas that of vendor A was preserved (82.6% versus 85.6%, *p* = 0.38). Consequently, accuracy for pleural effusion favored vendor B on PA radiographs (89.6% versus 86.8%, *p* = 0.003) but vendor A on portable AP radiographs (78.0% versus 66.8%, *p* < 0.001).

## 4. Discussion

This study demonstrates that two commercially available AI tools exhibit variable diagnostic performance across a range of chest abnormalities, when evaluated in a CT-anchored, CXR-targeted framework. Overall, specificity values were high, yet sensitivity and precision varied widely depending on the abnormality, highlighting the nuanced capabilities and limitations of each tool. Accuracy was high for both tools across all abnormalities and discriminated less clearly between them, reflecting the predominance of reference-negative instances in each analysis set; the practical differences between the systems are better conveyed by sensitivity, specificity, and precision.

As expected, chest X-ray has inherent technical limitations that constrain lesion visibility, particularly for small or obscured findings. In our study, nodules and atelectasis had the highest rates of X-ray invisibility, mostly due to size and location, while pulmonary edema was the most visible abnormality. These findings are consistent with prior reports [28,29,30,31,32]. Only 55.4% of CT-confirmed abnormalities were radiographically visible, underscoring that CT confirmation and radiographic detectability are not equivalent endpoints. Our primary analysis was therefore intentionally designed to evaluate AI performance for CT-confirmed abnormalities that were actually detectable on the paired CXR rather than for all abnormalities present on CT. This distinction is clinically relevant because CXR AI systems are deployed to interpret projection radiographs, not to recover findings that are not expressed on the radiograph itself. These visibility limitations affect both human readers and AI systems and should therefore be interpreted primarily as a constraint of the imaging modality rather than of the algorithm alone.

Vendor A demonstrated significantly higher sensitivity than vendor B for CT-confirmed, radiographically visible fractures, nodules, and pleural effusion. In contrast, vendor B showed higher specificity than vendor A across these categories—reaching 92% for nodules, 94% for pleural effusion, and 100% for fractures—along with higher precision for nodules and pleural effusion. Vendor B achieved numerically higher sensitivity for pneumothorax, although the difference was not statistically significant. Conversely, vendor B had significantly lower specificity, accuracy, and precision. However, the number of CT-confirmed pneumothorax cases was small, and precision was markedly low for both vendors. Therefore, these findings should be interpreted with caution and as exploratory. The performance metrics for detecting airspace disease were not significantly different between vendors. Since this harmonized category comprises distinct entities—atelectasis, opacity/consolidation, edema—for fair comparison between vendors with different annotation categories, lack of significance should not be interpreted as evidence of similar performance. While 56.6% of the airspace disease category adjudicated as visible on CXR were opacities/consolidations, harmonizing these distinct entities into a single group may have masked pathology-specific variations and resulted in a lack of statistical significance.

These findings underscore the variability in diagnostic performance between AI tools, depending on the abnormality. This variability mirrors prior studies, which report wide sensitivity ranges—33–96% for nodules, 72–98% for opacities, 54–82% for atelectasis, and 60–100% for pleural effusion and pneumothorax [5,6,9,12,13,23,30,33,34]. However, direct comparison with prior literature should be made cautiously because the reference paradigms differ substantially across studies. Most previous studies relied on radiologist reports or expert consensus as the endpoint. In contrast, our study used a CT-anchored, CXR-targeted framework that required both CT confirmation and radiographic visibility on the paired chest radiograph.

Prior CT-informed work also supports the importance of reference definition. Kim et al. reported different performance when the same algorithm was evaluated against CT-ground truth versus visible or clear-visible CXR ground truths, underscoring that strict CT positivity and radiographic detectability are distinct endpoints [17]. Likewise, Niehoff et al. established ground truth by consensus after review of the clinically anchored CXR interpretation rather than a purely CT-positive disease endpoint [23]. Accordingly, the present results should be interpreted as evaluating CXR AI under a CT-anchored radiographic-detectability paradigm.

Visibility adjudication with knowledge of the CT findings may nevertheless have been influenced by hindsight bias, in which prior knowledge of the outcome affects the interpretation of the original examination. Littlefair et al. reported that radiologists with outcome knowledge demonstrate significantly higher location-based sensitivity than blinded readers [35]. Kim et al. observed a similar pattern: when adjudicators annotated abnormalities on CXR with reference to CT, very subtle lesions were labeled that were difficult to identify on CXR without CT guidance, and the same algorithm showed a sensitivity of 69.6% against the CT-based ground truth compared with 82.7% against a visible CXR ground truth derived from consensus CXR readings, with the highest performance against a clear-visible ground truth (AUC 0.77, 0.84, and 0.87, respectively) [17]. Our CT-informed visibility adjudication is more inclusive of subtle findings than a ground truth derived from CXR-only readings and therefore occupies an intermediate position between these two reference definitions. Using all CT-confirmed abnormalities as reference-positive, without visibility adjudication, would have yielded the lowest sensitivity estimates, because findings with no radiographic manifestation would have been counted as false negatives. Visibility adjudication reduced but did not eliminate this effect: readers with access to CT are likely to have classified some subtle findings as visible that would not be confidently identified in routine CXR interpretation, where CT correlation is unavailable, and such findings are also less likely to be detected by AI. The reported sensitivity values should therefore be regarded as conservative estimates relative to the routine radiographic detectability of these abnormalities and would be expected to be higher against a blinded or clear-visible reference. This effect is likely to be more pronounced for subtle or equivocal findings, such as small nodules or minimal effusions, than for overt findings such as large pleural effusions or displaced fractures. Hindsight bias is not expected to have affected specificity or precision, because reference-negative status was determined by the absence of the abnormality on CT rather than by radiographic interpretation, and because the same reference set was applied to both tools, it affects the absolute estimates rather than the head-to-head comparison.

There are several studies that used CT as the reference in assessing the performance of AI solutions. For example, Bennani et al. analyzed 522 lesions using thoracic radiologists’ annotations based on CT exams and reported sensitivities for nodule, consolidation, pleural effusion, and pneumothorax were 60.3%, 81.5%, 89% and 88.6%, respectively. They stated that the use of CT to define reference standard may have resulted in a cohort consisting of more complex or doubtful cases that required further CT assessment [9]. Similarly, including only the patients who underwent CT, especially in a tertiary referral center, may have yielded a higher prevalence of pathology compared to the general population in our study, potentially influencing performance metrics and limiting the generalizability. Therefore, our precision values are likely overestimated compared to lower-prevalence settings. Another explanation might be our per-abnormality approach, in which multiple abnormalities of the same type in different regions, as well as different abnormality types within the same CXR, were analyzed separately. Lind Plesner et al. reported lower AI specificity for X-rays with multiple findings compared to those with single findings [30].

Performance also depended on radiographic projection. Both tools showed lower specificity for airspace disease and lower accuracy for pleural effusion on portable AP than on PA radiographs, consistent with the reduced technical quality, magnification and frequent suboptimal positioning of bedside imaging; this constraint applies to human readers as well and should be interpreted primarily as a limitation of the acquisition setting rather than of the algorithms. More notably, the two tools were not equally affected. The sensitivity of vendor B for pleural effusion fell by more than 25 percentage points on portable AP radiographs, whereas that of vendor A was largely preserved. This has direct implications for deployment, because the settings in which portable radiography predominates—intensive care, emergency and postoperative units—are also those in which the timely recognition of pleural effusion carries the greatest clinical weight. Institutions evaluating these tools should therefore consider the projection mix of their own workload rather than relying on pooled performance estimates alone.

While AI offers clear benefits in terms of detection and workflow support, it may also introduce the risk of false-positive findings as evidenced by relatively low precision values in the present work. Particularly for pneumothorax, vendor B’s very low precision (4%) at the default operating threshold suggests a potential false-positive burden in this cohort. In a real-world clinical setting, such performance may increase the risk of alert fatigue, unnecessary confirmatory imaging, and reduced clinician trust. However, given the low number of pneumothorax cases in this study, the performance metrics have limited statistical reliability and should not be overgeneralized. Likewise, in a study by Hwang et al., only 14.8% of AI-flagged abnormal X-rays were clinically relevant, with 85.2% representing false positives [29]. In another work, Hong et al. reported that the implementation of AI increased CT referral rates, including those with ultimately negative findings [36]. This emphasizes the need for a careful balance between sensitivity and specificity.

The variability of diagnostic performance across different AI tools depending on the abnormality underlines the importance of appropriate AI tool selection. In our study, the differences between the AI tools may reflect the differences in their training and validation datasets, and different annotation categories. Threshold selection is an important determinant of AI performance. As reported by Lind Plesner et al., sensitivity, specificity, and false-discovery rates of commercial CXR AI systems can vary substantially across different operating thresholds [30]. In the present study, both vendors were evaluated using the manufacturers’ default operating thresholds to reflect routine clinical use and to preserve head-to-head comparability. Site-specific recalibration was not feasible because probability scores and user-adjustable threshold parameters were not available for both tools. Although threshold optimization might improve performance for selected abnormalities, recalibrating only one tool—or applying non-equivalent recalibration strategies—would have reduced objectivity and comparability. Nonetheless, threshold optimization and site-specific calibration according to institutional needs are essential to improve overall performance and balance the trade-off between sensitivity and specificity.

High sensitivity thresholds may enhance the detection of subtle abnormalities, which is crucial for conditions where early recognition may alter triage or treatment, including airspace disease, pneumothorax, pleural effusion, and fracture. However, this trade-off can increase false-positive rates. For airspace disease, institutions should interpret performance relative to its intended use, because the harmonized category may include pulmonary edema, infectious consolidation, and atelectasis. Conversely, prioritizing specificity reduces unnecessary follow-ups but risks missing early or subtle disease. This is particularly important when AI outputs can trigger downstream imaging or procedural workup, as in pulmonary nodules, pleural effusions, and pneumothorax [10,36]. Therefore, AI tool selection should not be based solely on pooled accuracy or a single summary metric. There is no universally optimal operating point; the preferred balance between sensitivity and specificity depends on the target abnormality, disease prevalence, workflow integration model, and the institution’s tolerance for false-positive alerts. In emergency departments, intensive care units, or postoperative settings, a more sensitive tool may be preferable when AI is used for triage or worklist prioritization of time-sensitive findings and rapid human confirmation is available. In oncology practices or nodule-enriched populations, higher sensitivity for pulmonary nodules may be desirable to reduce missed potentially malignant lesions. However, lower specificity may lead to an increased number of false-positive findings, prompting further diagnostic workup with CT, causing unnecessary radiation exposure and anxiety for both patients and clinicians. By contrast, in lower-prevalence outpatient settings, higher specificity may be more valuable to limit unnecessary downstream imaging and false-positive workup [6,10,30,36].

Our study has several limitations. First, it was a retrospective single-center analysis performed at a tertiary referral center, and the CT-paired design enriched the cohort for abnormal and complex cases; therefore, the findings may not fully generalize to lower-prevalence outpatient, screening, or community-hospital populations. Second, although the maximum interval between chest radiography and CT was 14 days, temporal changes may still have occurred; to mitigate this, a stricter 2-day interval was applied for rapidly evolving findings. Third, our dataset included both PA and portable AP radiographs (19%). The inclusion of both projections better reflects our heterogeneous clinical setting. We acknowledge that AP projections have intrinsic technical limitations that may affect image quality and performance metrics of AI tools. To address this, we performed a subgroup analysis by radiographic projection (Supplementary Table S4). Fourth, because the two AI tools did not provide fully matched annotation taxonomies, vendor-specific airspace labels were harmonized into a single airspace disease category for fair comparison. This improved comparability but reduced pathology-specific granularity within the broader airspace spectrum. Since this category comprises distinct entities—such as atelectasis and consolidations—the performance metrics may primarily reflect the vendors’ general ability to detect lung opacification and should be interpreted with caution. Also, harmonization may have masked possible vendor-specific differences in detecting specific pathologies. Therefore, the lack of a significant difference between vendors for this category should not be interpreted as evidence of similar performance for each individual airspace pathology. Fifth, because the unit of analysis was the abnormality instance rather than the patient, the paired observations may not be fully independent. Cluster-robust and patient-level sensitivity analyses (Supplementary Tables S2 and S3) yielded concordant results, indicating that within-patient clustering did not drive the observed vendor differences; nevertheless, the patient-level analysis suggested small exploratory differences for harmonized airspace disease that were not evident at the lesion level, and estimates for pneumothorax remained unstable in both analyses because of the small number of positive cases. Sixth, both systems were evaluated at manufacturer-default operating thresholds, and site-specific threshold optimization could not be performed. Seventh, the software versions corresponded to the study period and may not reflect the performance of current versions as vendors continue to update training data, model calibration, and output behavior. While updated versions may yield more effective results and robust diagnostic performance, these dynamic changes may limit longitudinal comparability. Because vendor names and software versions cannot be disclosed, findings should be interpreted as reflecting AI performance in general rather than any specific vendor. Eighth, our CT-anchored, CXR-targeted design was intended to evaluate radiographic detectability rather than strict CT-defined disease burden; accordingly, the reported sensitivity values should be interpreted as sensitivity for CT-confirmed abnormalities that were radiographically detectable on the paired chest radiograph. Ninth, radiographic visibility was adjudicated with knowledge of the CT findings. Although visibility adjudication was intended to avoid penalizing the algorithms for findings with no radiographic manifestation, CT-informed readers may have classified some subtle findings as visible that would not be confidently identified in routine CXR interpretation; the reported sensitivity values are therefore likely to be conservative rather than inflated. Moreover, visibility assessment was performed in consensus rather than independently, and interobserver agreement statistics were therefore not calculated. Tenth, the markedly low number of CT-confirmed pneumothorax cases limits the reliability of performance comparison and the generalizability of these findings; therefore, performance metrics should be interpreted with caution. Finally, this study was designed as a comparison between two AI tools, and human reader performance was not included. Consequently, it was not possible to evaluate human reader performance or compare the performance of either tool with that of radiologists.

## 5. Conclusions

This study highlights that the diagnostic performance of different AI tools varies across abnormality categories when evaluated in a CT-anchored, CXR-targeted framework. Importantly, the airspace disease category is heterogeneous and harmonized, including different clinical entities such as atelectasis, opacity/consolidation, and pulmonary edema. Since these comparisons reflect the performance at each vendor’s default operating thresholds, the observed differences should not be interpreted as demonstrating vendor superiority but rather as evidence that AI tools differ in strengths across abnormality categories. Selection and clinical implementation of CXR AI should therefore be aligned with institutional priorities and the intended clinical use case. Future prospective multicenter studies involving different patient populations, disease prevalences, and institutional workflows are needed to assess the generalizability and robustness of AI performance across diverse clinical settings. Future studies should evaluate these tools after real workflow integration, including their effects on triage, reader performance, reporting time, missed-abnormality rates, and downstream consequences such as additional imaging, invasive procedures, and overall resource utilization.

## Figures and Tables

**Figure 1:**
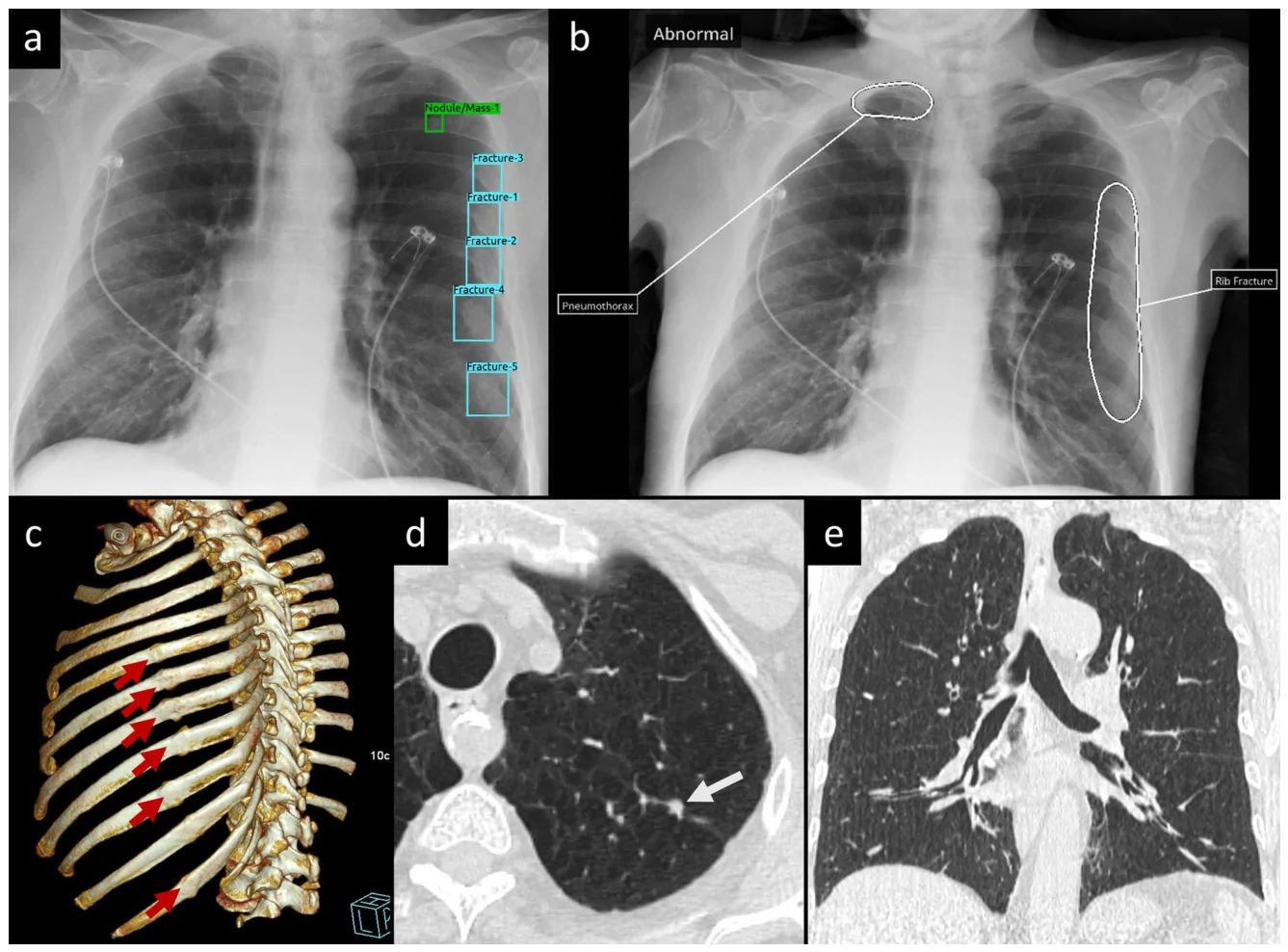
A patient with a suspicion of pneumonia. (**a**) Vendor A and (**b**) vendor B accurately identified left rib fractures. (**c**) 3D volume-rendered technique CT image better delineates the fractures (arrows). (**d**) Axial chest CT image shows a nodule (arrow) in the left upper lobe, which is correctly flagged by vendor A (**a**). (**e**) There is no pneumothorax on the coronal multiplanar reformat CT image, yet vendor B incorrectly flagged it as positive (**b**).

**Figure 2:**
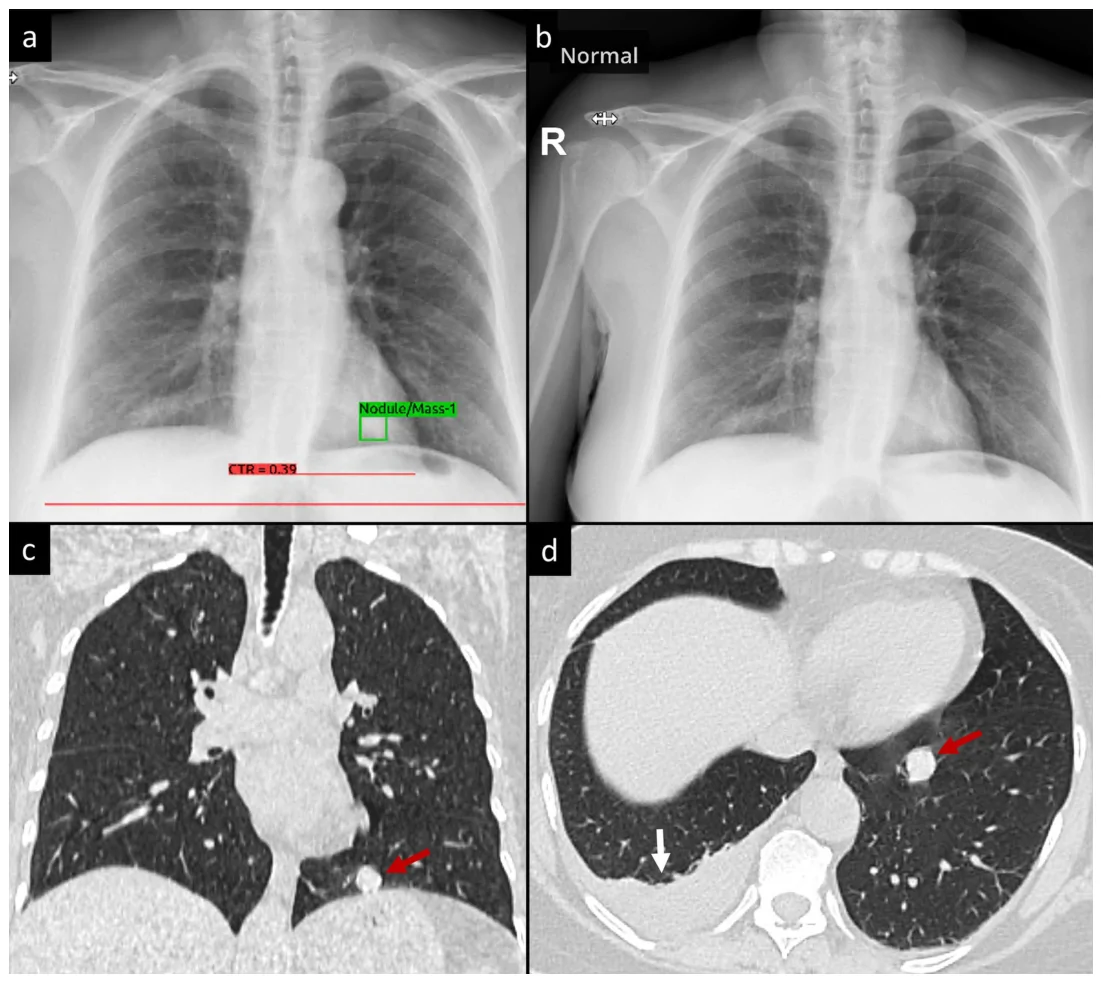
A patient with a known diagnosis of uterine leiomyosarcoma. (**a**) Vendor A accurately detected the retrocardiac nodule. (**b**) The nodule was not identified by vendor B, leading to a false negative result. On (**c**) coronal multiplanar reformat and (**d**) axial chest CT images, the nodule in the left lower lobe is seen (red arrows). Although present on CT (**d**, white arrow), the right pleural effusion is not visible on chest X-ray performed 7 days later (because this interval exceeded the study’s inclusion window, this finding was not included in the pleural effusion analysis).

**Figure 3:**
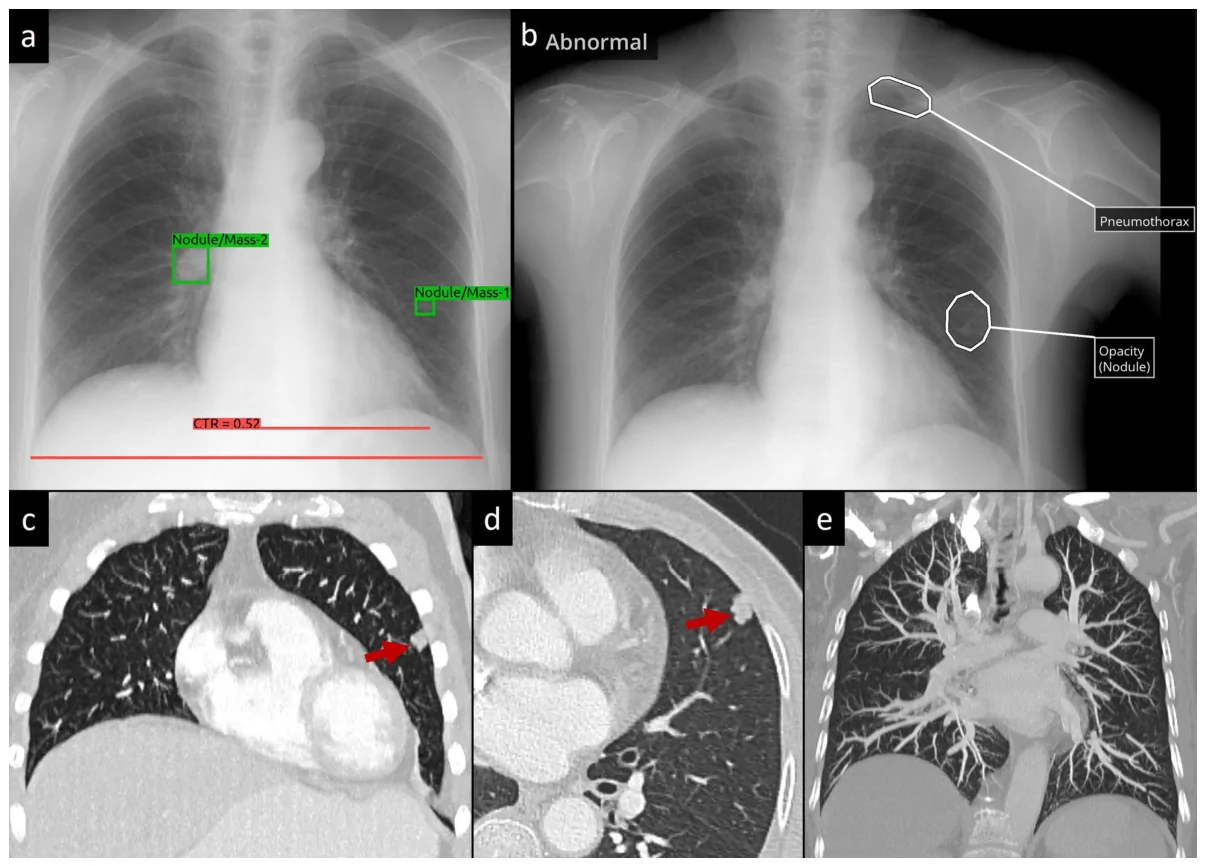
A patient with a known diagnosis of vulvar squamous cell carcinoma. (**a**) Vendor A and (**b**) vendor B correctly flagged the nodule located in the left lower zone. (**c**) Coronal maximum intensity projection (MIP) reformat and (**d**) axial CT images show the nodule in the lingula adjacent to the 4th intercostal space (arrows). Vendor A (**a**) incorrectly flagged a right hilar nodule/mass, and vendor B (**b**) incorrectly flagged a left apical pneumothorax, neither of which is seen on the coronal MIP reformat image (**e**).

**Figure 4:**
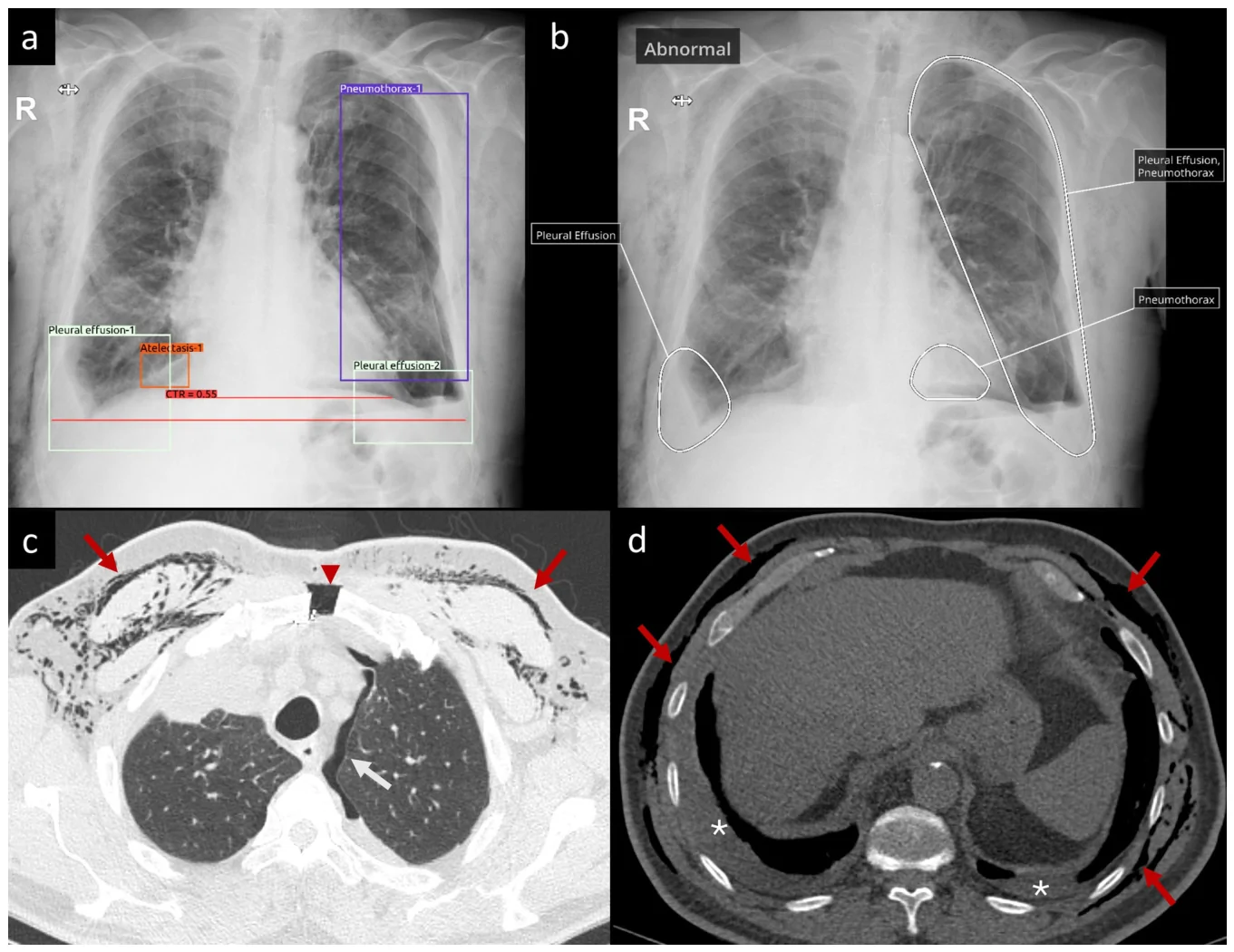
A patient who recently underwent coronary bypass surgery. (**a**) Vendor A and (**b**) vendor B correctly detected a left pneumothorax along with bilateral pleural effusion. Vendor A identified an atelectasis (orange bounding box) which was not detected by vendor B. Also, note the presence of subcutaneous emphysema. (**c**) Axial CT image confirms bilateral subcutaneous emphysema (red arrows) and left pneumothorax (white arrow) and also reveals sternal dehiscence (arrowhead). (**d**) Axial CT image demonstrates bilateral pleural effusion (asterisks) with subcutaneous emphysema extending to upper abdomen (arrows).

**Figure 5:**
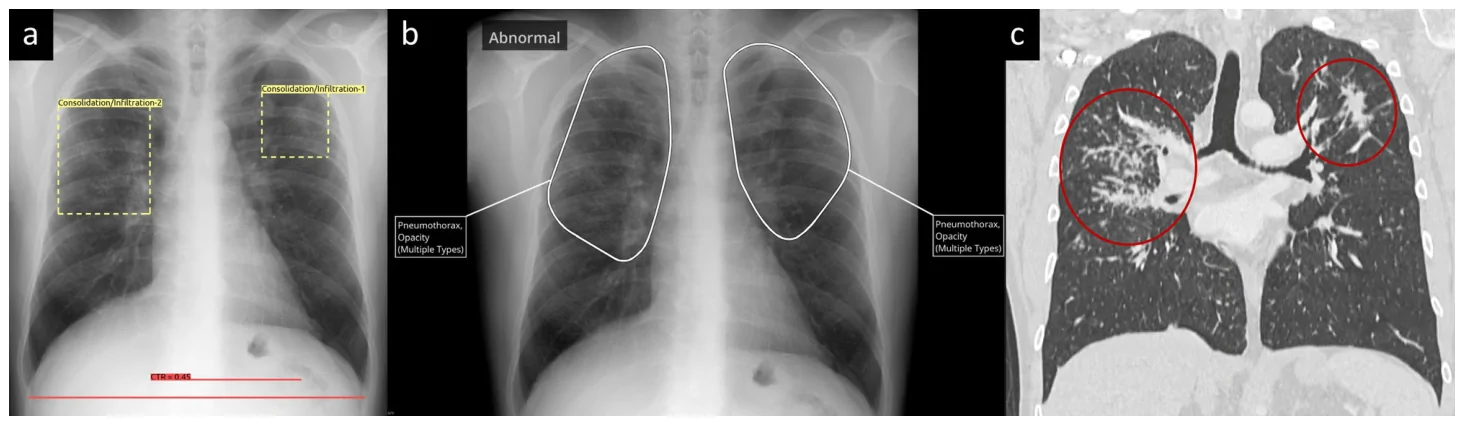
A patient with a known diagnosis of sarcoidosis. (**a**) Vendor A and (**b**) vendor B accurately identified bilateral opacities in the upper lung zones. (**c**) Coronal multiplanar reformat CT image demonstrates scattered peribronchovascular nodules, some of which are confluent in the upper lobes of both lungs (circles). Also, note that vendor B (**b**) incorrectly flagged pneumothorax in both lungs.

**Table 1:** Distribution of CT-confirmed abnormalities and chest X-ray visibility within the CT-anchored reference framework.

Pathology	Total AI-Labeled X-Ray Abnormalities (*n*)	RS-Negative (*n*)	RS-Positive (All) (*n*)	RS-Positive (Visible) (*n*)
Fracture	1836	1600	236	127
Nodule	1893	1110	783	195
Pleural effusion	1996	1322	674	482
Pneumothorax	1741	1715	26	20
Airspace disease	5692	3838	1854	1157

**Table 2:** Abnormality-based performance metrics of two different AI tools.

	Analysis set (*n*)	RS-Positive (Visible) (*n*)	RS-Negative (*n*)	Sensitivity, % (95% CI)	Specificity, % (95% CI)	Precision % (95% CI)	Accuracy, % (95% CI)
**Fracture**	1727	127	1600				
Vendor A				76 (68–83)	99 (98–99)	84 (77–90)	97 (96–98)
Vendor B				28 (21–36)	100 (99–100)	84 (70–92)	94 (93–95)
*p* value				<0.001	0.035	0.92	<0.001
**Nodule**	1305	195	1110				
Vendor A				71 (64–77)	87 (85–89)	49 (43–55)	85 (82–86)
Vendor B				50 (43–57)	92 (90–93)	52 (45–59)	86 (83–87)
*p* value				<0.001	<0.001	0.47	0.40
**Pleural effusion**	1804	482	1322				
Vendor A				84 (81–88)	85 (83–87)	68 (64–71)	85 (83–87)
Vendor B				61 (56–66)	94 (92–95)	78 (73–82)	85 (83–87)
*p* value				<0.001	<0.001	<0.001	1.000
**Pneumothorax**	1735	20	1715				
Vendor A				60 (39–78)	99 (98–99)	38 (23–55)	98 (97–99)
Vendor B				85 (64–95)	78 (76–80)	4 (3–7)	78 (76–80)
*p* value				0.063	<0.001	<0.001	<0.001
**Airspace disease**	4995	1157	3838				
Vendor A				63 (60–65)	93 (92–94)	72 (69–75)	86 (85–87)
Vendor B				65 (62–68)	93 (92–94)	74 (71–76)	86 (86–87)
*p* value				0.28	0.62	0.41	0.23

CI: Confidence interval; RS: reference standard. Sensitivity was calculated using only CT-confirmed abnormalities adjudicated as radiographically visible on the paired chest radiograph. CT-positive radiographically inapparent findings were excluded from the primary sensitivity analysis.

**Table 3:** Sensitivity and specificity of each AI tool across different abnormalities.

	Fracture		Nodule		Pleural Effusion		Pneumothorax		Airspace Disease	
	A	B	A	B	A	B	A	B	A	B
Analysis set (*n*)	1727		1305		1804		1735		4995	
RS-positive (visible)(*n*)	127		195		482		20		1157	
RS-negative (*n*)	1600		1110		1322		1715		3838	
Sensitivity (%)	**76**	28	**71**	50	**84**	61	60	**85**	63	**65**
Specificity (%)	99	**100**	87	**92**	85	**94**	**99**	78	93	93

RS: reference standard. For each abnormality, higher sensitivity and specificity values were presented in bold.

**Table 4:** Confusion matrix for both AI tools across different X-ray abnormalities.

Pathology	Vendor A				Vendor B			
	TP (*n*)	FP (*n*)	FN (*n*)	TN (*n*)	TP (*n*)	FP (*n*)	FN (*n*)	TN (*n*)
Fracture	97	18	30	1582	36	7	91	1593
Nodule	139	146	56	964	98	92	97	1018
Pleural effusion	407	195	75	1127	296	85	186	1237
Pneumothorax	12	20	8	1695	17	383	3	1332
Airspace disease	724	278	433	3560	750	267	407	3571

TP: True positive, FP: False positive, FN: False negative, TN: True negative. True-positive and false-negative counts are based only on CT-confirmed abnormalities adjudicated as radiographically visible on the paired chest radiograph. CT-positive radiographically inapparent findings were excluded from the primary analysis.

## Data Availability

The datasets presented in this article are not publicly available due to ethical and privacy restrictions and are subject to institutional regulations.

## Supplementary Materials

The following supporting information can be downloaded at https://www.mdpi.com/article/10.3390/diagnostics16193139/s1: Table S1: Multiple-comparison sensitivity analysis for primary head-to-head performance comparisons; Table S2: Cluster-robust (patient-clustered) re-analysis of the primary lesion-level head-to-head comparisons; Table S3: Patient-level sensitivity analysis (one observation per patient per abnormality category); Table S4: Head-to-head performance stratified by radiographic projection; Table S5: STARD-AI reporting checklist; Supplementary Figure S1. Flow diagram of the CT-anchored, CXR-targeted reference framework.

## Abbreviations

The following abbreviations are used in this manuscript:

AI	Artificial intelligence
AP	Anteroposterior
CE	Conformité Européenne (European Conformity)
CI	Confidence interval
CT	Computed tomography
CXR	Chest X-ray
DICOM	Digital Imaging and Communications in Medicine
FDA	U.S. Food and Drug Administration
FN	False negative
FP	False positive
IQR	Interquartile range
Lung-RADS	Lung CT Screening Reporting and Data System
MDR	European Union Medical Device Regulation
PA	Posteroanterior
PACS	Picture archiving and communication system
SD	Standard deviation
TN	True negative
TP	True positive
