# Supplementary material for: Diagnostic Performance of Different AI Tools for Chest Radiography: A CT-Anchored Per-Abnormality Analysis in a Tertiary Referral Center
Source: Diagnostics (Basel). 2026 Sep 27;16(19):3139. doi: 10.3390/diagnostics16193139 (PMC13664475; doi:10.3390/diagnostics16193139)
Supplement: Supplementary file 1 [file diagnostics-16-03139-s001.zip › Supplementary Table_S1.pdf]

Supplementary Table S1. Multiple-comparison sensitivity analysis for primary head-to-head performance comparisons

Abnormality	Metric	Vendor A, %	Vendor B, %	Raw p-value	Bonferroni-adjusted p-value	Holm-Bonferroni-adjusted p-value
**Fracture**	Sensitivity	76.4	28.3	**<0.001**	**<0.001**	**<0.001**
	Specificity	98.9	99.6	**0.035**	0.694	0.347
	Precision	84.3	83.7	0.92	1	1
	Accuracy	97.2	94.3	**<0.001**	**<0.001**	**<0.001**
**Nodule**	Sensitivity	71.3	50.3	**<0.001**	**<0.001**	**<0.001**
	Specificity	86.8	91.7	**<0.001**	**<0.001**	**<0.001**
	Precision	48.8	51.6	0.47	1	1
	Accuracy	84.5	85.5	0.40	1	1
**Pleural effusion**	Sensitivity	84.4	61.4	**<0.001**	**<0.001**	**<0.001**
	Specificity	85.2	93.6	**<0.001**	**<0.001**	**<0.001**
	Precision	67.6	77.7	**<0.001**	**<0.001**	**<0.001**
	Accuracy	85.0	85.0	1	1	1
**Pneumothorax**	Sensitivity	60.0	85.0	0.063	1	0.562
	Specificity	98.8	77.7	**<0.001**	**<0.001**	**<0.001**
	Precision	37.5	4.2	**<0.001**	**<0.001**	**<0.001**
	Accuracy	98.4	77.8	**<0.001**	**<0.001**	**<0.001**
**Airspace disease**	Sensitivity	62.6	64.8	0.28	1	1
	Specificity	92.8	93.0	0.62	1	1
	Precision	72.3	73.7	0.41	1	1
	Accuracy	85.8	86.5	0.23	1	1

Raw p-values are from the primary analysis. Bonferroni and Holm-Bonferroni corrections were applied across all 20 abnormality-by-metric comparisons. Sensitivity, specificity, and accuracy comparisons were based on paired comparisons; precision comparisons used the corresponding comparison of positive predictive values. Findings significant only before correction should be interpreted as exploratory.
