# Supplementary material for: Diagnostic Performance of Different AI Tools for Chest Radiography: A CT-Anchored Per-Abnormality Analysis in a Tertiary Referral Center
Source: Diagnostics (Basel). 2026 Sep 27;16(19):3139. doi: 10.3390/diagnostics16193139 (PMC13664475; doi:10.3390/diagnostics16193139)
Supplement: Supplementary file 1 [file diagnostics-16-03139-s001.zip › Supplementary Table_S2.pdf]

Supplementary Table S2. Cluster-robust (patient-clustered) analysis of the primary lesion-level head-to-head comparisons

Abnormality	Metric	n	Vendor A, % (95% CI)	Vendor B, % (95% CI)	p, primary paired test	p, patient-clustered
**Fracture**	Sensitivity	127	76.4 (68.3–82.9)	28.3 (21.2–36.7)	**<0.001**	**<0.001**
	Specificity	1600	98.9 (98.2–99.3)	99.6 (99.1–99.8)	**0.035**	**0.030**
	Precision	115 / 43	84.3 (76.6–89.9)	83.7 (70.0–91.9)	—	0.92
	Accuracy	1727	97.2 (96.3–97.9)	94.3 (93.1–95.3)	**<0.001**	**<0.001**
**Nodule**	Sensitivity	195	71.3 (64.6–77.2)	50.3 (43.3–57.2)	**<0.001**	**<0.001**
	Specificity	1110	86.8 (84.7–88.7)	91.7 (89.9–93.2)	**<0.001**	**<0.001**
	Precision	285 / 190	48.8 (43.0–54.6)	51.6 (44.5–58.6)	—	0.47
	Accuracy	1305	84.5 (82.5–86.4)	85.5 (83.5–87.3)	0.40	0.42
**Pleural effusion**	Sensitivity	482	84.4 (80.9–87.4)	61.4 (57.0–65.7)	**<0.001**	**<0.001**
	Specificity	1322	85.2 (83.2–87.1)	93.6 (92.1–94.8)	**<0.001**	**<0.001**
	Precision	602 / 381	67.6 (63.8–71.2)	77.7 (73.2–81.6)	—	**<0.001**
	Accuracy	1804	85.0 (83.3–86.6)	85.0 (83.3–86.6)	1.00	0.96
**Pneumothorax**	Sensitivity	20	60.0 (38.7–78.1)	85.0 (64.0–94.8)	0.063	**0.016**
	Specificity	1715	98.8 (98.2–99.2)	77.7 (75.6–79.6)	**<0.001**	**<0.001**
	Precision	32 / 400	37.5 (22.9–54.7)	4.2 (2.7–6.7)	—	**<0.001**
	Accuracy	1735	98.4 (97.7–98.9)	77.8 (75.7–79.6)	**<0.001**	**<0.001**
**Airspace disease**	Sensitivity	1157	62.6 (59.7–65.3)	64.8 (62.0–67.5)	0.28	0.32
	Specificity	3838	92.8 (91.9–93.5)	93.0 (92.2–93.8)	0.61	0.62
	Precision	1002 / 1017	72.3 (69.4–74.9)	73.7 (71.0–76.4)	—	0.41
	Accuracy	4995	85.8 (84.8–86.7)	86.5 (85.5–87.4)	0.23	0.30

Confidence intervals are Wilson score intervals. For pneumothorax sensitivity, the patient-clustered p-value is based on 20 reference-positive instances and is unstable at this sample size; the exact paired test is the primary result.
