# Supplementary material for: Diagnostic Performance of Different AI Tools for Chest Radiography: A CT-Anchored Per-Abnormality Analysis in a Tertiary Referral Center
Source: Diagnostics (Basel). 2026 Sep 27;16(19):3139. doi: 10.3390/diagnostics16193139 (PMC13664475; doi:10.3390/diagnostics16193139)
Supplement: Supplementary file 1 [file diagnostics-16-03139-s001.zip › Supplementary Table_S3.pdf]

**Supplementary Table S3. Patient-level sensitivity analysis (one observation per patient per abnormality category)**

| Abnormality | Patients (n) | Reference-positive (n) | Metric | Vendor A, % (95% CI) | Vendor B, % (95% CI) | p |
|---|---|---|---|---|---|---|
| **Fracture** | 1625 | 73 | Sensitivity | 78.1 (67.3–86.0) | 23.3 (15.1–34.2) | **<0.001** |
| | | | Specificity | 99.0 (98.4–99.4) | 99.5 (99.1–99.8) | 0.12 |
| | | | Precision | 79.2 (68.4–86.9) | 70.8 (50.8–85.1) | 0.35 |
| | | | Accuracy | 98.1 (97.3–98.7) | 96.1 (95.1–97.0) | **<0.001** |
| **Nodule** | 1130 | 134 | Sensitivity | 76.9 (69.0–83.2) | 46.3 (38.0–54.7) | **<0.001** |
| | | | Specificity | 90.3 (88.3–92.0) | 95.3 (93.8–96.4) | **<0.001** |
| | | | Precision | 51.5 (44.6–58.3) | 56.9 (47.5–65.8) | 0.17 |
| | | | Accuracy | 88.7 (86.7–90.4) | 89.5 (87.5–91.1) | 0.48 |
| **Pleural effusion** | 1565 | 332 | Sensitivity | 91.3 (87.7–93.8) | 60.5 (55.2–65.7) | **<0.001** |
| | | | Specificity | 87.1 (85.1–88.9) | 95.5 (94.1–96.5) | **<0.001** |
| | | | Precision | 65.6 (61.1–69.8) | 78.2 (72.8–82.8) | **<0.001** |
| | | | Accuracy | 88.0 (86.3–89.5) | 88.1 (86.4–89.6) | 1.00 |
| **Pneumothorax** | 1653 | 20 | Sensitivity | 60.0 (38.7–78.1) | 85.0 (64.0–94.8) | 0.063 |
| | | | Specificity | 98.8 (98.1–99.2) | 78.9 (76.8–80.8) | **<0.001** |
| | | | Precision | 37.5 (22.9–54.7) | 4.7 (3.0–7.4) | **<0.001** |
| | | | Accuracy | 98.3 (97.6–98.8) | 78.9 (76.9–80.8) | **<0.001** |
| **Airspace disease** | 1316 | 716 | Sensitivity | 79.6 (76.5–82.4) | 74.9 (71.6–77.9) | **0.028** |
| | | | Specificity | 89.3 (86.6–91.6) | 93.7 (91.4–95.4) | **0.002** |
| | | | Precision | 89.9 (87.3–92.0) | 93.4 (91.0–95.1) | **0.005** |
| | | | Accuracy | 84.0 (82.0–85.9) | 83.4 (81.3–85.3) | 0.68 |

*Confidence intervals are Wilson score intervals. The differences observed for airspace disease were not prespecified and are exploratory; the sensitivity difference would not remain significant after correction for multiple comparisons.*
