# Supplementary material for: Diagnostic Performance of Different AI Tools for Chest Radiography: A CT-Anchored Per-Abnormality Analysis in a Tertiary Referral Center
Source: Diagnostics (Basel). 2026 Sep 27;16(19):3139. doi: 10.3390/diagnostics16193139 (PMC13664475; doi:10.3390/diagnostics16193139)
Supplement: Supplementary file 1 [file diagnostics-16-03139-s001.zip › Supplementary Table_S4.pdf]

Supplementary Table S4. Head-to-head performance stratified by radiographic projection

Abnormality	Projection	n (RS+ / RS−)	Sensitivity A / B (p)	Specificity A / B (p)	Precision A / B (p)	Accuracy A / B (p)
**Fracture**	PA	1384 (98 / 1286)	75.5 / 24.5 **(<0.001)**	99.0 / 99.6 (0.08)	85.1 / 82.8 (0.75)	97.3 / 94.3 **(<0.001)**
	Portable AP	343 (29 / 314)	79.3 / 41.4 **(<0.001)**	98.4 / 99.4 (0.45)	82.1 / 85.7 (0.78)	96.8 / 94.5 (0.10)
**Nodule**	PA	1043 (174 / 869)	71.8 / 47.7 **(<0.001)**	85.4 / 90.7 **(<0.001)**	49.6 / 50.6 (0.81)	83.1 / 83.5 (0.82)
	Portable AP	262 (21 / 241)	66.7 / 71.4 (1.00)	92.1 / 95.4 (0.10)	42.4 / 57.7 (0.12)	90.1 / 93.5 (0.09)
**Pleural effusion**	PA	1436 (292 / 1144)	85.6 / 72.3 **(<0.001)**	87.2 / 94.1 **(<0.001)**	63.0 / 75.6 **(<0.001)**	86.8 / 89.6 **(0.003)**
	Portable AP	368 (190 / 178)	82.6 / 44.7 **(<0.001)**	73.0 / 90.4 **(<0.001)**	76.6 / 83.3 (0.09)	78.0 / 66.8 **(<0.001)**
**Pneumothorax**	PA	1390 (15 / 1375)	60.0 / 86.7 (0.12)	99.4 / 76.7 **(<0.001)**	52.9 / 3.9 **(<0.001)**	99.0 / 76.8 **(<0.001)**
	Portable AP	345 (5 / 340)	60.0 / 80.0 (1.00)	96.5 / 81.8 **(<0.001)**	20.0 / 6.1 **(0.001)**	95.9 / 81.7 **(<0.001)**
**Airspace disease**	PA	3924 (710 / 3214)	58.6 / 63.1 (0.09)	94.8 / 94.4 (0.39)	71.5 / 71.3 (0.95)	88.3 / 88.7 (0.48)
	Portable AP	1071 (447 / 624)	68.9 / 67.6 (0.72)	82.1 / 86.1 (0.05)	73.3 / 77.6 (0.14)	76.6 / 78.3 (0.34)
