# Supplementary material for: Diagnostic Performance of Different AI Tools for Chest Radiography: A CT-Anchored Per-Abnormality Analysis in a Tertiary Referral Center
Source: Diagnostics (Basel). 2026 Sep 27;16(19):3139. doi: 10.3390/diagnostics16193139 (PMC13664475; doi:10.3390/diagnostics16193139)
Supplement: Supplementary file 1 [file diagnostics-16-03139-s001.zip › Supplementary Table_S5.pdf]

Supplementary Table S5. STARD-AI reporting checklist

Section and topic	No.	STARD-AI item	Reported	Location
**Title or abstract**				
	1	Identification as a study reporting AI-centered diagnostic accuracy and reporting at least one measure of accuracy within title or abstract	Yes	Title
**Abstract**				
	2	Structured summary of study design, methods, results and conclusions (for specific guidance, see STARD for Abstracts)	Yes	Abstract
**Introduction**				
	3	Scientific and clinical background, including the intended use of the index test, whether it is novel or an established index test and its integration into an existing or new workflow, if applicable	Yes	Introduction
	4	Study objectives and hypotheses	Yes	Introduction
**Methods**				
**Study design**	5	Prospective or retrospective data collection	Yes	Materials and Methods-Study population
**Ethics**	6	Formal approval from an ethics committee.	Yes	IRB statement
**Participants**	7	Eligibility criteria: listing separate inclusion and exclusion criteria	Yes	Materials and Methods-Study population
	8	On what basis potentially eligible participants were identified	Yes	Materials and Methods-Study population
	9	Where and when potentially eligible participants were identified (setting, location and dates)	Yes	Materials and Methods-Study population
	10	Whether participants formed a consecutive, random or convenience series	Yes	Materials and Methods-Study population
**Dataset**	11	Source of the data and whether they have been routinely collected, specifically collected for the purpose of the study or acquired from an open-source repository	Yes	Materials and Methods-Study population
	12	Who undertook the annotations for the dataset and how	Yes	Materials and Methods-Abnormality-based X-ray evaluation and CT-anchored reference framework
	13	Devices (manufacturer and model) that were used to capture data; software (with version number) used to engineer the index test, highlighting the intended use	Partial	Materials and Methods-Data collection, AI software used in the study; Discussion-Limitations
	14	Data acquisition protocols (for example, contrast protocol or reconstruction method for medical images) and details of data preprocessing, in sufficient detail to allow replication	Yes	Materials and Methods-Data collection, AI software used in the study
**Test methods**	15a	Index test, in sufficient detail to allow replication	Partial	Materials and Methods-AI software used in the study

	15b	How the index test was developed, including any training, validation, testing and external evaluation, detailing sample sizes, when applicable	Yes	Materials and Methods-AI software used in the study
	15c	Definition of and rationale for test positivity cutoffs or result categories of the index test, distinguishing prespecified from exploratory	Yes	Materials and Methods-AI software used in the study
	15d	The specified end-user of the index test and the level of expertise required of users	No	
	16a	Reference standard, in sufficient detail to allow replication	Yes	Materials and Methods-Abnormality-based X-ray evaluation and CT-anchored reference framework
	16b	Rationale for choosing the reference standard (if alternatives exist)	Yes	Introduction, Materials and Methods
	16c	Definition of and rationale for test positivity cutoffs or result categories of the reference standard	Yes	Materials and Methods-Abnormality-based X-ray evaluation and CT-anchored reference framework
	17a	Whether clinical information and reference standard results were available to the performers or readers of the index test	Yes	Materials and Methods-AI software used in the study
	17b	Whether clinical information and index test results were available to the assessors of the reference standard	Yes	Materials and Methods-Abnormality-based X-ray evaluation and CT-anchored reference framework
**Analysis**	18	Methods for estimating or comparing measures of diagnostic accuracy	Yes	Materials and Methods-Statistical analysis
	19	How indeterminate index test or reference standard results were handled	Yes	Materials and Methods-Abnormality-based X-ray evaluation and CT-anchored reference framework
	20	How missing data on the index test and reference standard were handled	Yes	Materials and Methods-AI software used in the study
	21	Any analyses of variability in diagnostic accuracy, distinguishing prespecified from exploratory	Yes	Materials and Methods-Statistical analysis
	22	Intended sample size and how it was determined	Yes	Materials and Methods-Statistical analysis
	23	Details of any performance error analysis and algorithmic bias and fairness assessments, if undertaken	Partial	Materials and Methods-Statistical analysis; Results
**Results**				
**Participants and dataset**	24	Flow of participants, using a diagram	Partial	Supplementary Figure S1
	25	Baseline demographic, clinical and technical characteristics of training, validation and test sets, if applicable	Yes	Results
	26a	Distribution of severity of disease in those with the target condition	No	
	26b	Distribution of alternative diagnoses in those without the target condition	No	

	27	Time interval and any clinical interventions between index test and reference standard	Yes	Materials and Methods-Study population
	28	Whether the datasets represent the distribution of the target condition that one would expect from the intended use population	Yes	Materials and Methods-Study population; Discussion
	29	For external evaluation on an independent dataset, an assessment of how this differs from the training, validation and test sets	No	
**Test results**	30	Cross-tabulation of the index test results (or their distribution) by the results of the reference standard	Yes	Results-Table 4
	31	Estimates of diagnostic accuracy and their precision (such as 95% confidence intervals)	Yes	Results-Table 2,3, S1-S3
	32	Any adverse events from performing the index test or the reference standard	NA	
**Discussion**				
	33	Study limitations, including sources of potential bias, statistical uncertainty and generalizability	Yes	Discussion- Limitations
	34	Implications for practice, including the intended use and clinical role of the index test	Yes	Discussion; Conclusions
	35	Ethical considerations and adherence to ethical standards associated with the use of the index test and issues of fairness	Yes	Discussion
**Other information**				
	36	Registration number and name of registry	No	
	37	Where the full study protocol can be accessed	No	
	38	Sources of funding and other support; role of funders	Yes	Funding statement
	39	Commercial interests, if applicable	Yes	Conflict of Interest statement
	40a	Availability of datasets and code, detailing any restrictions on their reuse and repurposing	Yes	Data Availability statement
	40b	Whether outputs are stored, auditable and available for evaluation, if necessary	Yes	Methods – AI Software Used in the Study
